# Leveraging global tools for adolescent and youth health planning and programming: process and lessons learnt from Madagascar

**DOI:** 10.1080/16549716.2026.2641326

**Published:** 2026-03-26

**Authors:** Anaclet Ngabonzima, Sarah C. Keogh, Regina Guthold, Lethicia Lydia Yasmine, Geoffrey K. Bisoborwa, Sehenolalao Andrianasolo, Eugenie Siga Diane Niane, Maurice Ye, Miary Toky Rajoelina, Navalona Rakotomahefa Fetrarivo, Laurent Musango

**Affiliations:** a World Health Organization Country Office (WCO), Antananarivo, Madagascar; b World Health Organization, Geneva, Switzerland; c Ministry of Health, Antananarivo, Madagascar; d World Health Organization Regional Office for Africa (AFRO), Brazzaville, Congo; e Department of Public Health, Free University of Brussels, Brussels, Belgium

**Keywords:** Adolescent health, AA-HA! guidance, GAMA-recommended indicators, multi-sectoral collaboration, evidence-based planning

## Abstract

Madagascar, with a significant proportion of its population aged 10–24 years, has developed a comprehensive National Strategic Plan for Adolescent and Youth Health (2025–2030) to improve adolescent and youth health and well-being. This process applied the second edition of Accelerated Action for the Health of Adolescents (AA-HA!) guidance and the Global Action for Measurement of Adolescent Health (GAMA)-recommended indicators to ensure an evidence-based approach. Moving beyond the traditional focus on sexual and reproductive health, the strategy adopted a multi-sectoral approach that involved government ministries, non-governmental organizations (NGOs), and youth associations, among other stakeholders. Key steps included stakeholder engagement, establishing a steering committee, mapping and analyzing data for GAMA-recommended indicators, assessing health needs, reviewing policies, and setting priorities through participatory, data-driven methods. Findings revealed major challenges, including high rates of adolescent fertility and early marriage, thinness among adolescent girls, and substance abuse. Integrating GAMA-recommended indicators with adolescent perspectives provided a holistic view of health needs, enabling the development of an inclusive strategic plan. Lessons learned highlight the importance of robust data use, multi-sectoral collaboration, and active youth involvement in planning and programming. Madagascar’s experience demonstrated that applying AA-HA! guidance and GAMA-recommended indicators is essential for a comprehensive approach to adolescent and youth health planning and programming. This process identified real needs and informed evidence-based strategies, reinforcing the value of inclusive, data-driven planning for improving health outcomes among adolescents and youth.

## PAPER CONTEXT

- **Main findings:** Madagascar developed a comprehensive National Strategic Plan for Adolescent and Youth Health for 2025–2030 using the global guidance on Accelerated Action for the Health of Adolescents and the Global Action for Measurement of Adolescent Health indicators.
- **Added knowledge:** This experience shows how global frameworks for adolescent and youth health can be effectively applied at country level to create holistic strategies.
- **Global health impact for policy and action:** Madagascar’s approach provides a replicable model for countries aiming to strengthen adolescent and youth health planning.

## Background

As a second sensitive developmental window following early childhood, adolescence represents an optimal period in life for learning healthy behaviours, acquiring social and labour skills, and realizing human rights protections that have an impact throughout the life course [1–6]. In fact, a considerable number of key risk factors for diseases and conditions that develop later in adulthood are linked to adolescence [7,8]. Therefore, investment made in this second decade of life delivers the ‘triple dividend’ of improving health and well-being now, enhancing it throughout the life course, and contributing to the health and well-being of future generations [9–11]. Achieving the Sustainable Development Goals (SDGs), including universal health coverage (SDG Target 3.8), requires maintaining adolescents’ health and well-being so they can survive and thrive now and into adulthood, as recognized in the Global Strategy for Women’s, Children’s and Adolescents’ Health [12]. However, to fulfil the promises of the SDGs, further support and commitment are required to deliver for adolescents, especially those most at risk of being left behind [13]. Ensuring that every adolescent can make informed choices about their lives and fulfil their rights to attain full health and well-being requires more than just engaging them in planning processes that affect them. It also requires moving beyond the health sector and developing robust multisectoral, whole-of-government policy approaches that address adolescent health and well-being holistically [14,15].

Adolescents face many health risks, including nutritional problems, mental health issues, violence, and substance abuse, which can have long-term consequences [8,16–18]. However, adolescent health issues have often been overlooked in public health debates, as many view this life stage as a period of generally good health. This perception results in a lack or fewer targeted interventions and services tailored for adolescents, even though they encounter significant health challenges during this period [19]. In many countries, including Madagascar, adolescent health planning and programming have been largely focused on sexual and reproductive health and rights (SRHR) at the expense of other important aspects [20,21]. There is increasing awareness of the importance of addressing adolescents’ health needs holistically, beyond SRHR [13,20,22]. In addition to their health being overlooked, for many years, adolescents have been viewed mainly through protective, beneficiary, or instrumentalist lenses and not necessarily seen as rights-holders [14]. As a result, planning and programming for adolescents’ health have overlooked their perspectives, which has limited improvements in their health [14,20,23].

To overcome this challenge, the World Health Organization (WHO) and other partners developed the Global Accelerated Action for the Health of Adolescents (AA-HA!) guidance to equip governments to respond to the health and well-being challenges, opportunities and needs of adolescents with a view to better operationalize the adolescent component of the Global Strategy for Women’s, Children’s and Adolescents’ Health (2016–2030) [12,24,25]. To track progress towards health improvements, the Global Action for Measurement of Adolescent Health (GAMA)-recommended indicators, published in 2024, assess a wide range of adolescent health issues. Available data on the indicators can be used to improve adolescent health planning and programming by supporting the identification of priorities, the allocation of resources, the benchmarking of progress, and the evaluation of policies and programs, among other uses [26–28].

In Madagascar, as in many countries of sub-Saharan Africa, the population is young. According to the 2018 Third General Population and Housing Census (RGPH-3), Madagascar has a population of 25.7 million inhabitants, approximately 50% of whom are under 20 years old. Adolescents aged 10–19 years constitute approximately 23% of the total population, while youth aged 15–24 represent approximately 20%. The country’s population continues to increase at an annual growth rate of 3% [29]. To ensure the next generation is healthy and productive, the country must design and implement evidence-based interventions to improve the health and well-being of the Malagasy adolescents. Yet, as highlighted by the adolescent and youth strategic plan 2018–2020, adolescent health planning and programming have only focused on SRHR and overlooked other health components [30].

Recognizing that adolescent well-being depends on a range of factors, the Government of Madagascar adopted, in 2024, a more holistic approach with mutually reinforcing interventions across sectors. The new strategic plan aims to advance adolescent health and well-being through tackling all pressing health issues beyond reproductive health. To this end, in the second half of 2024, the Government of Madagascar, under the overall coordination of the Ministry of health and in collaboration with other governmental sectors such as education and social protection as well as non-governmental actors involved in adolescent health such as WHO, World Bank/GFF, UNICEF, UNFPA, and USAID, initiated the development of a new comprehensive National Strategic Plan (NSP) for Adolescent and Youth Health using the AA-HA! guidance version 2 and the Adolescent health and well-being data tool [31].

This manuscript presents the participatory, data-driven process following the AA-HA! guidance and using the GAMA-recommended indicators to develop Madagascar’s first-ever comprehensive adolescent and youth health strategy, enabling other countries undergoing similar processes to replicate elements and gain insights from this process.

## Methods

### Study design

This study employed a qualitative descriptive design, using a program-planning and process- documentation approach to document how global tools were applied in Madagascar’s adolescent and youth health strategic planning process.

### Strategic plan development steps

The development of the National Strategic Plan for Adolescent and Youth Health adopted an inclusive approach, involving a range of actors in accordance with AA-HA guidance. The process comprised several steps (Panel 1), which are described in more detail below.

**Panel 1.**
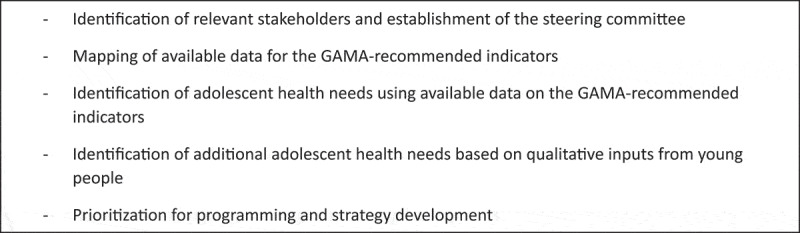
Steps followed to develop the National Strategic Plan for Adolescent and Youth Health.

### Identification of relevant stakeholders and establishment of the steering committee

Prior to initiating the process, the Ministry of Health organized a one‑day kickoff meeting, held at the Ministry of Health, to convene various financial and technical partners involved in adolescent health. The meeting provided an opportunity to agree on the overall process, the methodological approach, and the specific steps to be followed. Participants agreed on using the inclusive and data-driven approach and mapped out different organizations and actors to get involved in the process. The organizations, structures and actors identified included ten (10) ministries, namely the Ministry of health, Ministry of Population and Solidarity, Ministry of Water, Hygiene and Sanitation, Ministry of Youth and Sports, Ministry of National Education, Ministry of Technical Education and Vocational Training, Ministry of Public Security, Ministry of Tourism, Ministry of Justice, and Ministry in charge of the traffic police. It also included technical and financial partners such as the World Health Organization (WHO), World Bank/Global Financing Facility (WB/GFF), United Nations Children’s Fund (UNICEF), United Nations Population Fund (UNFPA), United States Agency for International Development (USAID), World Doctors Madagascar (MDM), and Marie Stopes Madagascar (MSM). Professional health associations were also involved, including the National Order of Doctors (ONM), National Order of Midwives (ONSFM), and National Order of Nurses (ONIM). In addition, civil society organizations such as the Lutheran Health Department (SALFA), the Malagasy Christian Church Health Network (FISA), and SCOUT participated, along with youth associations including the Network of Organizations of Youth and Women (NOSYW), African Youth and Adolescents Network (AfriYan), and Tanora Iray.

In addition to identifying various actors, the kickoff meeting established a steering committee to coordinate all activities throughout the process. The subsequent steps following this kickoff meeting, described below, involved those identified stakeholders.

### Mapping of available data for the GAMA-recommended indicators

The next step involved mapping all available data sources for the GAMA-recommended indicators, using the French version of the Adolescent Health and Well-Being Data Tool [32]. This step involved the Monitoring and Evaluation focal points or database managers from the aforementioned-mentioned stakeholder organizations. During the workshop, participants identified a range of relevant data sources, including recent surveys such as the Demographic and Health Survey (DHS), ongoing national strategies and policies, and other key sources. This mapping exercise was conducted over a three-day period in the capital city.

## Results

### Identified adolescent health needs through analysis of available data aligned with the GAMA-recommended indicators

The Global Action for Measurement of Adolescent Health (GAMA) is a collaborative initiative led by WHO and UN H6+ partners to strengthen and harmonize the measurement of adolescent health and well-being at global, regional, and national levels. Established in 2018, GAMA aims to address longstanding gaps and inconsistencies in adolescent health measurement by providing a comprehensive set of 47 indicators that span health outcomes, behaviors, determinants, and systems performance. These indicators are designed to guide evidence-based policy, programming, and monitoring efforts, ensuring that adolescent health is effectively tracked and prioritized in alignment with the Sustainable Development Goals [33].

Following the completion of the Adolescent health and well-being data tool through two workshops of two days each, the GAMA-related data were analyzed with the support of the World Health Organization (WHO). This analysis was key to identifying the most pressing issues for adolescent health in Madagascar and allowed comparison of the indicators against national and global benchmarks. Some results from the analysis are described in panel 2.

**Panel 2.**
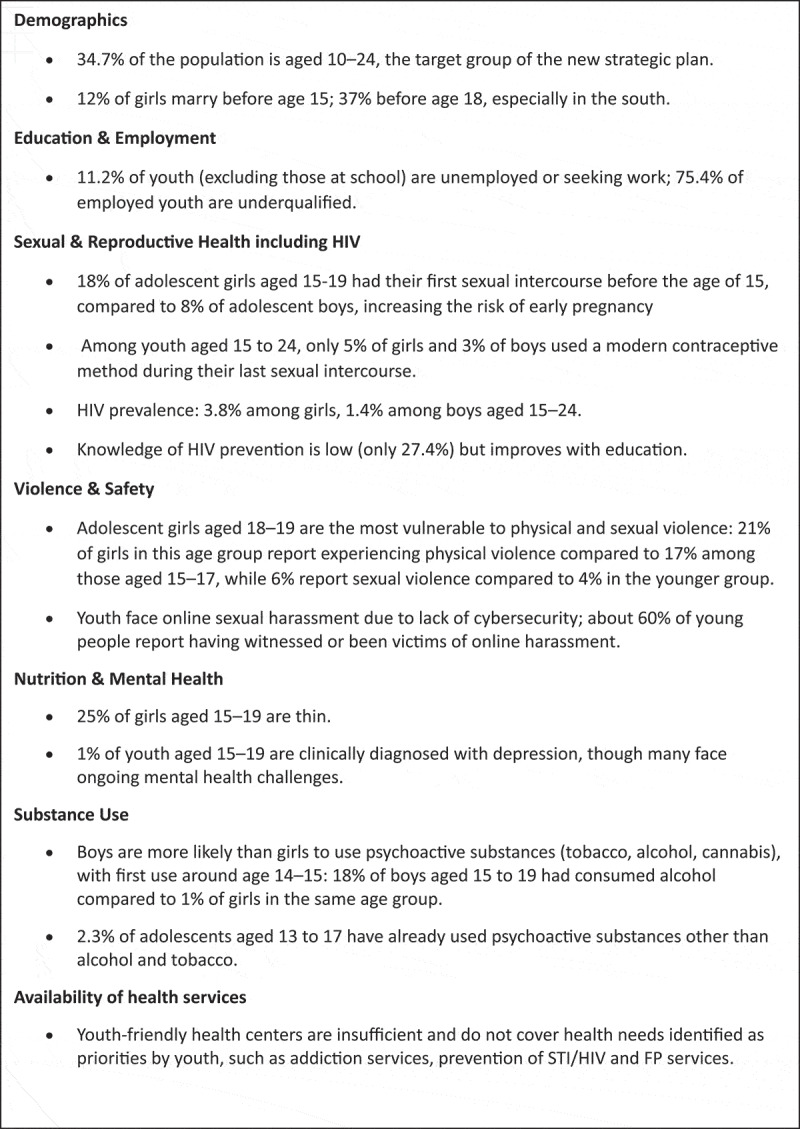
Key insights on adolescent and youth health in Madagascar [34,37,37].

**Figure 1.**
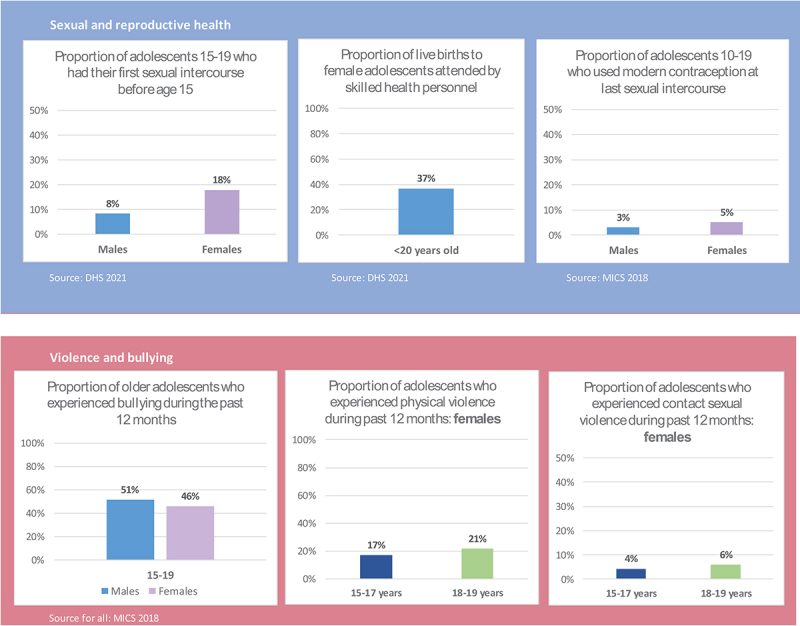
Example of outputs from analysis of GAMA- recommended indicators. Source: Madagascar National Strategic Plan for the Health of Adolescents and Youth 2025–2030.

Different outputs from the analysis of GAMA-recommended indicators were synthetized in the form of different figures to make the setting of priorities easier [31]. An example of the outputs is given in Figure 1.

### Identification of additional adolescent health needs based on qualitative inputs from young people

To ensure an inclusive approach in developing the National Strategic Plan for Adolescent and Youth Health, the youth association NosyW conducted guided focus group discussions with young people aged 15 to 24 across Madagascar’s 22 regions (out of 23). These discussions revealed the expectations and priorities of Malagasy youth aged 15 to 24 for optimized health services [31]. These expectations and priorities are summarized in Panel 3.

**Panel 3.**
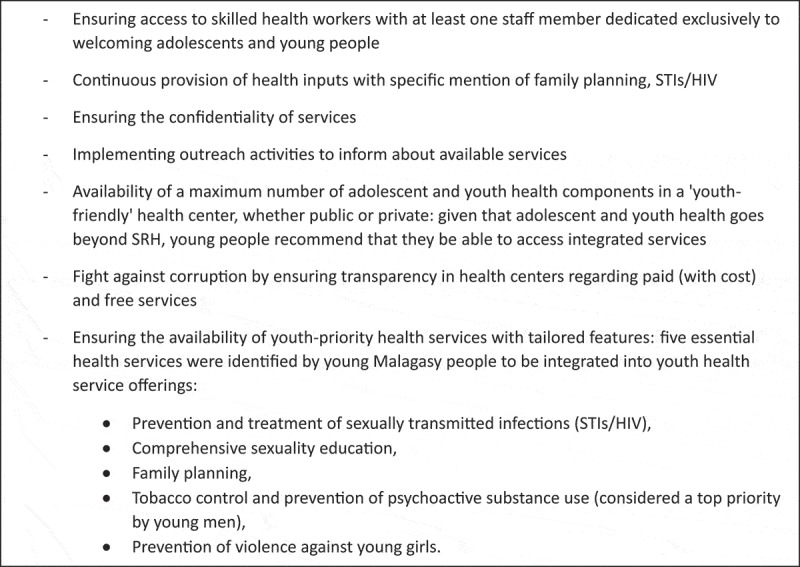
Expectations and priorities of Malagasy youth as per group discussions [31].

### Prioritization for programming and strategy development

The final step, ‘prioritization for programming and strategy development,’ was carried out during the Pre-Data to Action (PDTA) and Data to Action (DTA) workshops, which aimed to set priorities and design programs to key areas. The PDTA workshop was conducted over two days, followed by the four-day DTA workshop. Together, these activities informed and contributed to the development of the National Strategic Plan for Adolescent and Youth Health.

The strategy was initially developed by a consultant consultation with a restricted technical committee, followed by a one-week pre-validation workshop to ensure that the document accurately reflected the priorities established during the DTA workshop. This pre-validation workshop brought together various stakeholders, including youth associations from different regions of the country, many of whom had participated in the PDTA and DTA workshops.

A one-day validation and dissemination workshop was then held, gathering a wide range of actors involved in adolescent and youth health, under the leadership of the Ministry of Health. The development of the strategy was recognized as a key milestone in improving adolescent and youth health, as highlighted by senior government officials.

“I am delighted to see the Strategic Plan for Adolescent and Young People’s Health come to fruition, with contributions from various ministries. This unprecedented collaboration highlights our unified approach to addressing the diverse health needs of our youth,” said **Lethicia Lydia YASMINE, Secretary General of the Ministry of public health in Madagascar.**

The formulated activities were comprehensively summarized under five strategic axes (Panel 4).

**Panel 4.**
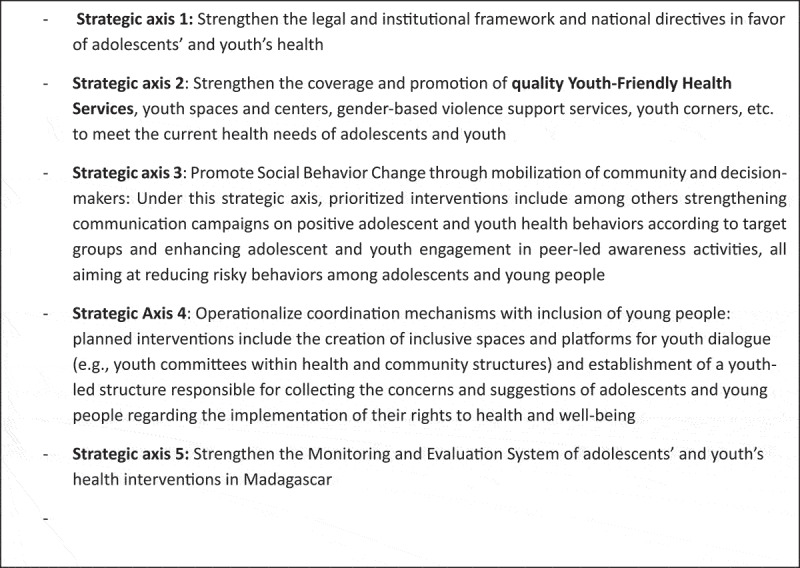
Five strategic axes of Malagasy adolescent and youth health strategy [31].

### From planning to action: key insights from early implementation

Following the validation of Madagascar’s first Adolescent and Youth Health Strategic Plan, the country initiated early implementation in 2025 through coordinated government actions and partner-supported initiatives. Key achievements included the development of an operational plan to guide phased implementation, the increasing awareness and capacity‑building initiatives to strengthen the delivery of adolescent- and youth‑friendly health services and setting up a structured mechanism to effectively share essential information with adolescents and young people. This mechanism involved the development and validation of content for e‑learning modules designed to improve access to standardized, evidence‑based training materials.

These initial achievements demonstrate a strong national commitment to advancing adolescent and youth health and represent meaningful progress toward achieving the strategy’s objectives. Collectively, they reflect a shift toward a more holistic and integrated approach to adolescent well‑being, moving beyond Madagascar’s earlier programming, which predominantly emphasized sexual and reproductive health.

## Discussion and lessons learned

In the next sections, we discuss Madagascar’s experience in developing the NSP for Adolescent and Youth Health using the AA-HA! guidance version 2 and the GAMA recommended indicators, including lessons learned, challenges, and examination of similar efforts in other countries.

### The use of AA-HA! guidance

The AA-HA! guidance offers a structured approach for countries to develop, implement, and update national strategies for adolescent health. It underscores the importance of investing in adolescents to achieve the SDGs and calls for coordinated, multisectoral efforts to address health determinants across all levels, from the individual to the structural [24,25].

Madagascar’s use of the AA-HA! approach aligns with these principles, ensuring that the strategic plan is evidence-based and comprehensive. The AA-HA! guidance encourages countries to adopt a menu of interventions and implementation strategies tailored to their specific context, rather than a one-size-fits-all approach. This flexibility allows Madagascar to address its unique challenges and leverage its strengths in planning and programming for adolescent health. The experiences and challenges encountered during the planning process align with key components of the AA-HA! guidance, particularly those related to data collection, the complexity of multisectoral coordination, and meaningful youth engagement. These aspects are especially critical given the country’s vast geographical size and diverse population. These lessons learnt and challenges are described in the respective sections below [31].

Another major challenge has been the significant variation in priorities and issues affecting adolescents and youth across different regions of the country. In addition, cultural norms and beliefs that influence adolescent health vary across regions, necessitating tailored interventions. For example, menstruation remains a taboo subject in some rural areas, while it is more openly discussed in others. Cultural barriers in certain regions also prevent young girls from expressing their needs related to menstrual hygiene. To address these issues, Madagascar is prioritizing the involvement of community and traditional leaders to raise awareness, foster dialogue, and promote culturally sensitive approaches to adolescent and youth health [31].

### Use of multisectoral approaches

According to AA-HA guidance, comprehensive strategic planning and programming should be multisectoral and not the sole responsibility of the Ministry of Health. Indeed, interventions to address issues affecting adolescent health should bring together a variety of actors, including ministries (health, education, justice, social action, security, and civil protection) and non-governmental partners [24,25]. In Madagascar, the process involved the participation of ten (10) different ministries working with adolescents and youth in one way or another, which allowed for tackling the most pressing issues affecting adolescents and youth from a multisectoral lens. In addition, the involvement of law makers is key. In Madagascar, the national parliament accompanied the process through to its conclusion, raising the possibility of reforming certain laws that hinder adolescent health and increasing in budget allocations to adolescent health. Importantly, the process involved youth participation from the outset through to the final validation of the strategy (as described in more detail below). Involving different stakeholders, including youth, in planning processes ensured that their voices and perspectives were heard and valued, while also creating a space for collaboration and shared responsibility, resulting in solutions that better reflect community needs and preferences. Despite involving a diverse range of stakeholders as per AA-HA! guidance, the process was primarily attended by participants from central-level ministries. Given Madagascar’s vast geography, inviting all regional representatives would have posed significant logistical challenges. Additionally, when experts from various ministries come together for planning, each tends to focus first on activities specific to their own institution, which necessitate extended discussions and deliberations. Similar challenges may arise during implementation. To mitigate this, Madagascar has prioritized establishing a well-structured and robust multisectoral coordination platform at both national and subnational levels, ensuring inclusive participation, effective implementation, and accountability across all regions [31].

### More insights into the use of the GAMA-recommended indicators and the Adolescent health and well-being data tool

The use of Adolescent health and well-being data tool to analyze the status of the 47 GAMA-recommended *indicators* facilitates the measurement of adolescent health across six key domains: physical health, mental health, sexual and reproductive health, risk behaviors, social environment, and access to health services. These indicators provide a framework for countries to collect reliable, comparable data, which is key to identifying the most pressing issues and guiding policies and programs to improve adolescent health outcomes [32,33]. In Madagascar, the process of collecting data to populate the Adolescent health and well-being data tool brought together various stakeholders. It became evident that no single source can provide comprehensive data for all GAMA-recommended indicators. Importantly, the analysis facilitated access to age- and sex-disaggregated data, which are often lacking, making it difficult to use the data effectively for program planning. The GAMA-recommended indicators also draw much-needed attention to often marginalized subgroups and overlooked issues. For example, they enable measurement of out-of-school children and address data gaps in areas such as mental health, injury, bullying, and positive well-being indicators. However, while the Adolescent health and well-being data tool helped map available data of sufficient quality and coverage against the defined indicators, most existing sources still lack data for the 10–14 age group [31]. Current tools and assessments used in Madagascar should be expanded to include this critical age group.

### Use of a participatory approach including adolescent and youth

A key aspect of Madagascar’s experience in developing the NSP 2025–2030 was the active involvement of youth in the planning process. Firstly, youth associations themselves conducted group discussions with adolescents and youth across nearly all regions of Madagascar. This approach enabled the collection of authentic views on gaps and priorities directly from adolescents and youth. Such insights would have been difficult to obtain if the discussions had been led by adults. For example, one of the key wishes expressed by youth during these discussions was the fight against corruption. They emphasized the importance of ensuring transparency in health centers regarding paid and free services, to prevent discrimination or favoritism in healthcare facilities. This concern might not have been revealed as clearly if the discussions had not been youth-led.

Secondly, in addition to identifying youth needs, adolescents and young people participated in workshops and consultations throughout the process, sharing their perspectives and priorities. This inclusive approach not only empowered youth but also ensured that the strategic plan was relevant and responsive to their needs. The youth involvement fostered a sense of ownership and commitment among all parties, enhancing the likelihood of successful implementation and sustainability of the strategic plan, and upholding the standard of meaningful engagement of adolescents and youth in the services intended for them, as well as the principle of no longer doing in the place of users but with them as per AA-HA! guidance [24,25,31]. This type of involvement of Malagasy youth through their different associations should continue during the implementation phase of the strategic plan and should be distinguished from one-off actions that are not very productive.

### Role of the ministry of health leadership

The leadership of the Ministry of Health played a pivotal role in the successful development of Madagascar’s comprehensive NSP for Adolescent and Youth Health. By spearheading the coordination of the process, the Ministry ensured that adolescent health was elevated as a national priority and that the planning was inclusive, evidence-based, and aligned with global standards. The Ministry’s proactive engagement in convening stakeholders, establishing a multisectoral steering committee, and endorsing the use of AA-HA! guidance and GAMA recommended-indicators demonstrated strong institutional commitment. This leadership was instrumental in fostering collaboration across sectors such as education, social protection, and youth affairs and in mobilizing technical and financial support from key partners. Moreover, the Ministry’s endorsement of a holistic approach to adolescent health, beyond sexual and reproductive health, signaled a paradigm shift towards recognizing adolescents as rights-holders and active participants in shaping their health agenda. This strategic vision and stewardship were essential in driving the process forward and ensuring its relevance, sustainability, and alignment with national development goals.

### Comparison with experiences from other countries

To inform its approach, Madagascar explored the experiences of a few other countries that had adopted AA-HA! guidance in developing their National Adolescent Health Strategic Plans, such as Belize [35]. Like Belize, Madagascar’s process included a comprehensive needs assessment, data analysis, and participatory consultations with service providers and adolescents. As in Belize, Madagascar emphasized multi-agency and multi-sector collaboration, ensuring inputs from both national and subnational levels. In Belize, workshops with adolescents and service providers were conducted to review and validate the strategic plan, focusing on priority areas to improve adolescent health outcomes. This human rights-based and life cycle approach was applied in Madagascar to plan a health response that supports stage-specific interventions, promotes equity and inclusion, ensures continuity of care, and facilitates positive transitions to adulthood. Another key aspect highlighted in Belize’s experience and evident in Madagascar’s process is the strong government ownership. The process advanced smoothly because it was led and owned by the Ministry of Health, which also conducted formal outreach to other ministries and stakeholders.

Other experiences explored included those from Sri Lanka and Tunisia, both of which recently developed adolescent health strategies that employed rights-based and participatory approaches in line with AA-HA! guidance [36,37]. However, unlike Madagascar, their processes did not incorporate GAMA-recommended indicators, a distinctive feature of Madagascar’s approach.

It is worth noting that our review of literature did not identify *any* documentation or published evidence showing that any country to date has applied both AAHA! guidance and the GAMArecommended indicators in the development of a national adolescent health strategy. This absence is likely due to the fact that the GAMA-recommended indicators were only very recently published, officially released by WHO in 2024. Given the novelty of these indicators, global documentation on their practical use remains very limited.

This limited evidence base highlights the significance of Madagascar’s experience. As one of the first countries to operationalize the newly published GAMA-recommended indicators within the context of national adolescent health planning, Madagascar provides an early and practical example of how these indicators can be translated into policy and programmatic decisionmaking. For this reason, we consider it essential to document this process in detail. By sharing Madagascar’s experience, this manuscript offers valuable guidance for other countries that may soon undertake similar efforts as they begin to adopt and apply both AAHA! guidance and the GAMA indicator framework in their own strategic planning.

## Conclusion

Making the needs of adolescents and youth visible through the collection and analysis of data on GAMA-recommended indicators is an essential step to better understanding the health issues faced by this population group. This approach helps implement targeted interventions and actions that support their development and wellbeing, not only during adolescence, but throughout their adult lives. The experience in Madagascar showed that the use of AA-HA! guidance and GAMA-recommended indicators facilitated a holistic approach to programming, highlighting critical health issues and identifying the real needs of Malagasy adolescents and youth. This enabled evidence-based programming and the prioritization of key areas outlined in the AA-HA! guidance. Successful implementation of the developed strategy will require maintaining this multisectoral approach, engaging young people, and closely monitoring progress and accountability throughout the process. This will facilitate interventions that protect youth from health and social risks, support their mental well-being, and encourage their active participation in society, thereby contributing to achieving the SDG targets related to adolescent health.

## Data Availability

Datasets of Adolescent health and well-being data tool filled out as well as the full strategic plan are available for sharing if need be.
